# Minimally invasive subpial tonsillectomy for Chiari I decompression

**DOI:** 10.1007/s00701-016-2877-2

**Published:** 2016-07-05

**Authors:** Jeffrey S. Beecher, Yong Liu, Xiaoming Qi, Paolo A. Bolognese

**Affiliations:** 1 Department of Neurosurgery, UT Southwestern, 5323 Harry Hines Blvd, Dallas, TX 75390-8855 USA; 2 Yuquan Hospital, Department of Syringomyelia, Tsinghua University, 5 Shijingshan Rd Shijingshan District, Beijing, 100049 China; 3 Division of Neurosurgery, South Nassau Communities Hospital, 1 Healthy Way, Oceanside, NY 11572 USA

**Keywords:** Chiari I malformation, Posterior fossa decompression, Cerebellar tonsil resection, Minimally invasive subpial resection, Syringomyelia

## Abstract

**Background:**

A number of different surgical techniques have been used through the years to address Chiari I malformation (CMI).

**Methods:**

This article describes how we surgically manage CMI at two high-volume centers. We call the technique the minimally invasive subpial tonsillectomy (MIST). The technique consists of a minimalistic dissection and craniectomy with a short, linear durotomy for the subpial tonsillar resection. The dura is closed without the use of a duraplasty.

**Conclusions:**

We describe our current methods of surgery for CMI.

**Electronic supplementary material:**

The online version of this article (doi:10.1007/s00701-016-2877-2) contains supplementary material, which is available to authorized users.

## Background

Chiari I malformation (CMI) is defined by a small posterior fossa with herniation of the hindbrain through the foramen magnum [1–3]. A spectrum of surgical techniques has been utilized over the years to treat this disorder [2, 4–8]. All of them provide different degrees of decompression of the cervicomedullary junction. There remains controversy regarding which techniques are most effective in treating the pertinent anatomic pathology [2–6]. Some authors advocate for resection of the cerebellar tonsils, while others do not [3, 5, 9].

Our technique for CMI is called minimally invasive subpial tonsillectomy (MIST). The technique consists of a minimalistic dissection, a small craniectomy, a C1 laminotomy, and a midline durotomy with subpial resection of the cerebellar tonsils. The dura is closed primarily. This technique originated at the Department of Syringomyelia in Beijing, China.

## Methods

### Relevant surgical anatomy

CMI surgery is centered on the junction of the foramen magnum and the posterior arch of C1. The cerebellar tonsils, by definition, will have herniated through the foramen magnum and often displaced well below C1. It is imperative that dissection appropriately uncovers the most inferior aspect of the tonsils. This is confirmed with intraoperative ultrasound that also reveals the neighboring anatomy of the cervicomedullary junction and the mass effect upon it from the tonsils. Intradurally, the tonsillar loops of the posterior inferior cerebellar arteries (PICAs) are in the midline, and running laterally are the spinal accessory nerves that also warrant decompression in many cases.

### Description of the technique

**Fig. 1:**
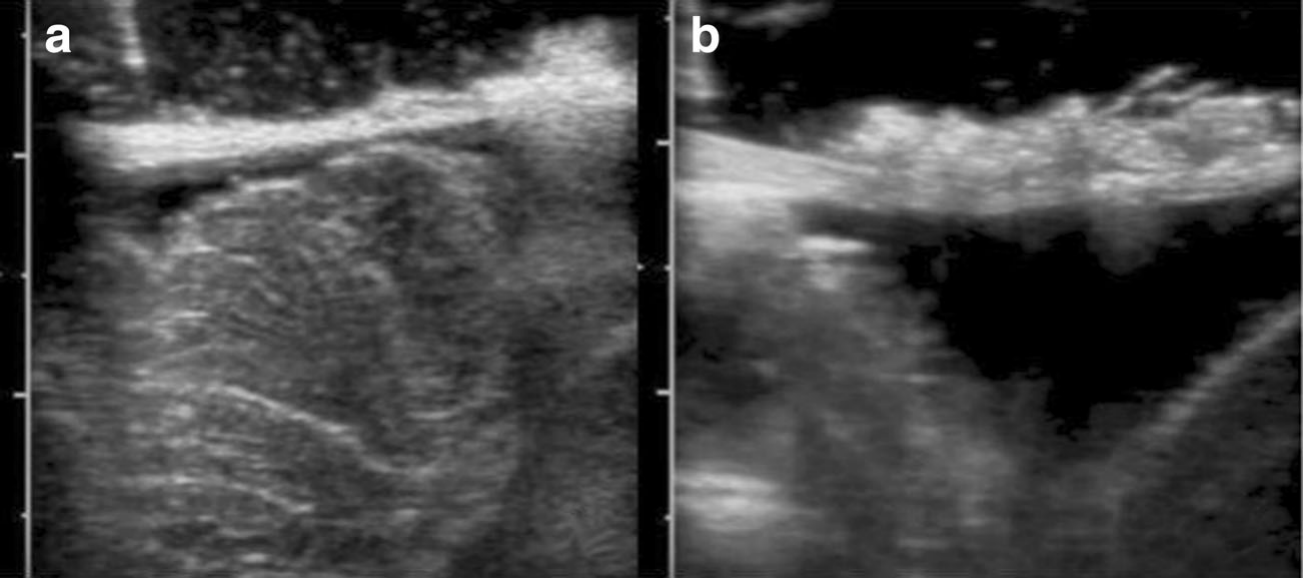
**a** Intraoperative ultrasound demonstrating the cerebellar tonsil prior to durotomy and subpial resection. **b** Intraoperative ultrasound after MIST is performed to confirm adequate tonsillar resection

The patient is positioned prone with the chin tucked with care not to kind the endotracheal tube, and the head is fixed with the Mayfield® skull clamp (Integra Life Sciences Corporation, Cincinnati, OH, USA). A 5–6½-cm midline skin incision is centered 1 cm above the craniocervical junction. Minimal electrocautery is used on the subcutaneous tissues, and is mainly reserved for the midline dissection of the linea alba. The narrow soft tissue dissection is followed with a small square-shaped suboccipital craniectomy 2 cm in length and 2–3 cm wide, followed by a C1 laminotomy (removal of the superior rim of the C1 lamina). A complete laminectomy is performed whenever the cerebellar tonsils are radiographically determined to be inferior to the arch of C1. Transdural intraoperative ultrasound is use to visualize the tonsils in the sagittal plane and this guides the durotomy length (Fig. 1a).

**Fig. 2:**
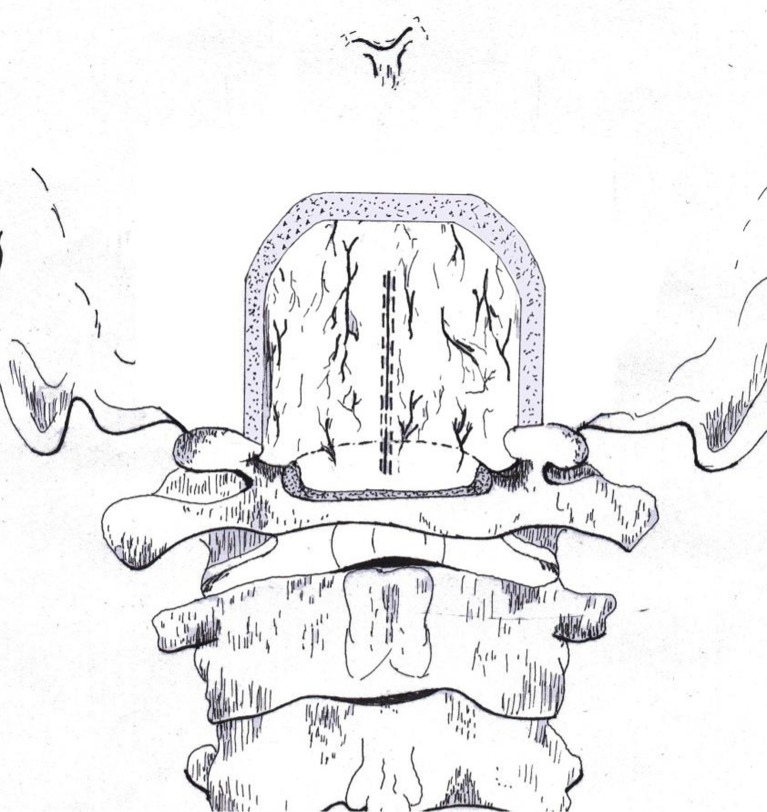
Artistic rendering of the bone work and linear durotomy for MIST

The linear, midline durotomy ranges between 2 and 3 cm, and is performed under the microscope (Fig. 2). The dural edges are retracted with sutures. Surgical visualization is increased by movements of the operative table, and with gentle retraction of the dura with a nerve hook. The arachnoid is incised and resected, and CSF is drained. The PICA tonsillar loops are dissected from the inferior aspect of the tonsils.

Arachnoid bands are dissected off the tonsils to allow for mobility, and bipolar coagulation is then initiated along their posterior, medial, and inferior aspects. A horizontal incision is made along the mid-portion of the tonsils with sharp dissection. The tonsillar parenchyma is coagulated while gently suctioning away debris. The dissection is performed in a medial to lateral fashion. A number of vertically arranged septations are encountered within the tonsils, and these require sharp dissection. The pial surface of the tonsils will become increasingly mobile, and this allows for dissection of the adhesions between the tonsils and the brainstem. The tonsils become progressively mobile and the lateral adhesions to the accessory nerve become accessible for dissection, while the tonsillar surface is more generously coagulated. The tonsillar resection is complete when: (1) Foramen of Magendie is unobstructed, (2) the cervicomedullary junction and the accessory nerves are decompressed, and (3) ¾ of the cerebellar tonsils are resected. This is confirmed with intraoperative ultrasound (Fig. 1b).

**Fig. 3:**
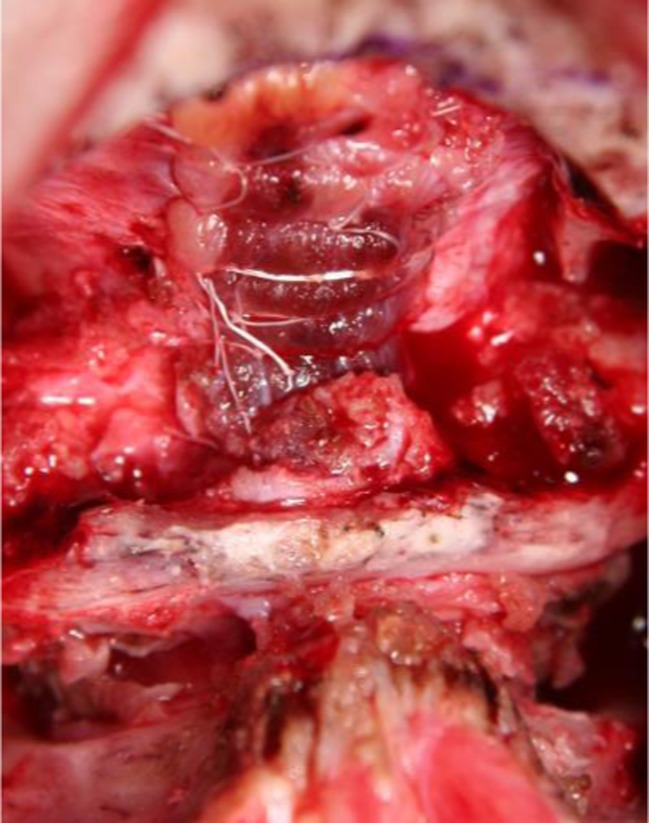
Muscle patch sutured over the dural closure

When possible, the pial edges surrounding the tonsillar incisions are re-approximated with a 7-0 Gore-Tex suture (Gore-Tex, W. L. Gore Inc., Flagstaff, AZ, USA). The dura is closed primarily with running unlocked 5-0 Gore-Tex sutures. A thin muscle patch is secured over the dural closure with 7-0 Gore-Tex interrupted sutures bridging over the muscle (Fig. 3). The anesthesia team provides Valsalva maneuvers to test the integrity of the closure (Video Supplement).

### Indications

The indications for surgery are the presence of one or more of the following criteria:

1. Karnofsky score of 70 or less secondary to Chiari malformation stereotypic constellation of symptoms
2. An expanding syringomyelia on consecutive MRI scans, syringomyelia cavities in excess of 75 % of the transverse cord diameter on the index MRI, or eccentric appearance of the syringomyelia cavity with intraparenchymal blebs
3. Severe, rapidly progressive neurological deficit

### Limitations

There is an inherently longer learning curve in achieving the surgical goals through the described minimalistic approach. It is important to note that the resection of the cerebellar tonsils does come with risk. The direct surgical manipulation and resection of the tonsils can lead to a number of possible complications such as cerebellar edema or ischemia, injury to PICA, hemorrhage, brainstem or spinal cord injury, and cranial nerve injuries.

### How to avoid complications

Above all, it is imperative that the dural edges are handled with care and the re-approximation is completed in a meticulous fashion. This, in combination with the muscle on-lay, dramatically reduces the risk of pseudomeningocele formation.

### Specific perioperative considerations

**Fig. 4:**
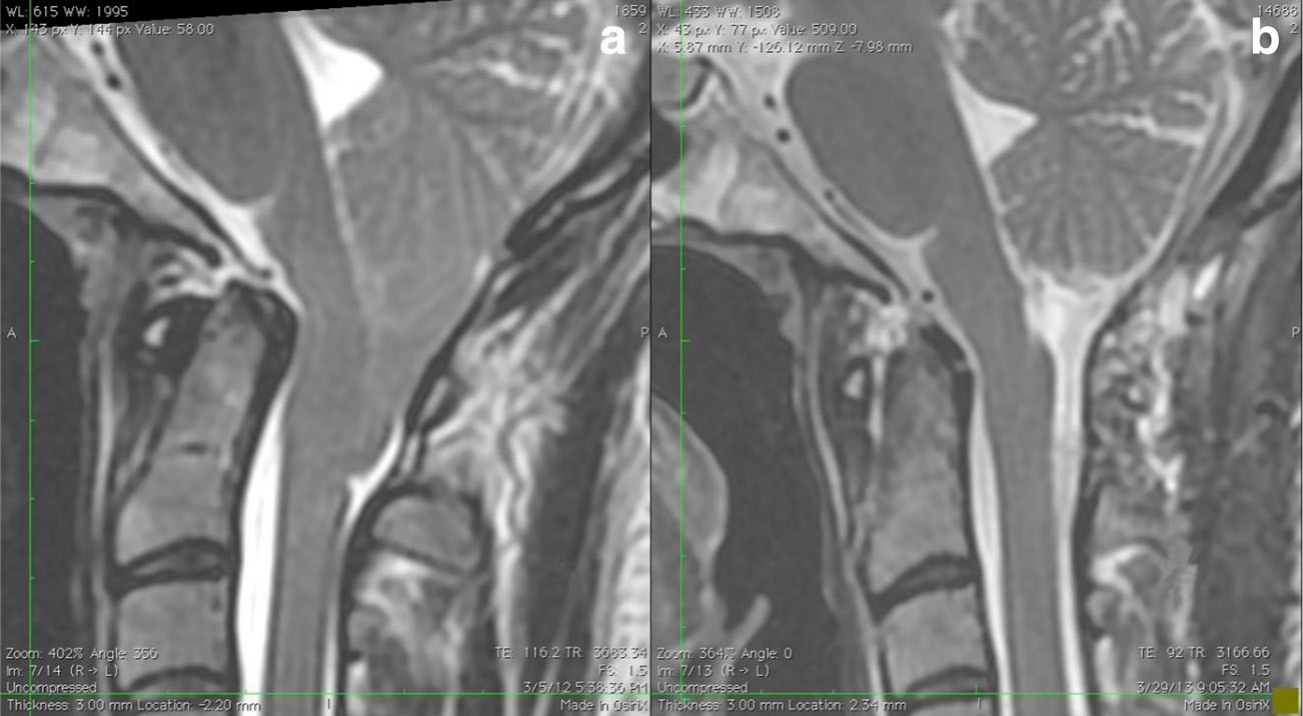
**a** Preoperative and **b** postoperative MRI of a MIST procedure and it should be noted this patient concomitantly underwent a craniocervical fusion for basilar invagination

Preoperatively, it is imperative to study the patient’s MRI and consider the length of the cerebellar tonsils herniation in relation to the posterior arch of C1 (Fig. 4a). This predicts how much of the C1 lamina will require removal to permit a useful durotomy.

Intraoperatively, aggressive subpial resection of the cerebellar tonsils provides direct decompression of the cervicomedullary junction as opposed to traditional techniques aimed at indirect decompression with excessive craniectomies or expansile duraplasties [3–6, 9]. Furthermore, the minimal disruption of the soft tissues and bone may have an impact on wound healing and possibly decreasing the number of patients who progress to require a craniocervical fusion. The MIST technique has decreased our revision rate, which is likely secondary to the intraoperative evaluation of the tonsil resection. This provides immediate feedback regarding the cervicomedullary decompression that is confirmed with intraoperative ultrasound. Prior to closure of the dura, the anesthesia team is asked to administer one dose of 25 g of mannitol intravenously. Lastly, a primary closure is utilized and decreases the risk of pseudomeningocele formation.

Postoperatively, a 24-h course of dexamethasone 4 mg intravenously every 6 h is prescribed. In combination with the intraoperative mannitol, this has been used to minimize any edema in the cerebellum from the surgery. The patient is monitored in the ICU for at least 24 h, and early mobilization is initiated on postoperative day 1along with neck mobilization. An MRI is obtained to confirm the extent of resection and is compared to the preoperative study (Fig. 4b).

Perioperatively, we have found a very low complication rate (Tables 1 and 2), and believe MIST leads to the ideal patient outcomes.

**Table 1:** Patient demographic information

General statistics	Beijing	NY
Time range	2004–2015	2011–2015
Females/males	969 (64.8 %) / 525 (35.2 %)	151 (83.8 %) / 29 (16.2 %)
Mean age ± SD	39.3 ± 10.8 SD	34.3 ± 11.7 SD
Age range	2–76	9–73
Pediatric/adults	38 (2.5 %) / 1,456 (97.5 %)	18 (10.0 %) / 162 (90.0 %)
Virgin/redo	1490 (99.7 %) / 4 (0.3 %)	139 (77.2 %) / 41 (22.8 %)
Total cases	**1**,**494**	**180**

**Table 2:** Complications

Complications	Beijing	NY
Hydrocephalus	7 (0.4 %)	0 (0.0 %)
Meningitis	3 (0.2 %)	0 (0.0 %)
Permanent bulbar palsy	4 (0.3 %)	0 (0.0 %)
Cerebellar infarction	3 (0.2 %)	0 (0.0 %)
Cerebellar hemorrhage	1 (0.1 %)	0 (0.0 %)
Pseudomeningocele	3 (0.2 %)	0 (0.0 %)
Total	**21** (**1.4** %)	**0** (**0.0** %)

### Specific information to give to the patient about surgery and potential risks

Emphasis is placed on the inherent risk with resection of the cerebellar tonsils. This is in addition to the standard risk profile of the traditional operation.

## Key points

- Preoperative MRI review for length of cerebellar tonsils
- Small craniectomy with minimal C1 lamina removal
- Midline, linear durotomy
- Maximize visualization of the cerebellar tonsils
- Subpial resection is performed from medial to lateral and is considered complete when:
  - Foramen of Magendie is unobstructed
  - No evidence of compression of the cervicomedullary junction
  - ∼ ¾ of the cerebellar tonsils have been resected
- Intraoperative ultrasound confirmation of surgical goals
- Primary closure of the dura with a muscle on-lay sutured in place

## Video Supplement

Below is the link to the electronic supplementary material.

ESM 1 This video demonstrates the step by step process of performing the MIST technique from skin incision to closure of the dura. (MP4 97604 kb)
